# Clinical Follow-Up without Radiographs Is Sufficient after Most Nonoperatively Treated Distal Radius Fractures in Children

**DOI:** 10.3390/children10020339

**Published:** 2023-02-09

**Authors:** Marja Perhomaa, Markus Stöckell, Tytti Pokka, Justus Lieber, Jaakko Niinimäki, Juha-Jaakko Sinikumpu

**Affiliations:** 1 Research Unit of Clinical Medicine, Medical Research Center, Oulu Childhood Fracture and Sports Injury Study, Division of Pediatric Surgery and Orthopedics, Department of Children and Adolescents, (MRC) Oulu, Oulu University Hospital, Oulu University, 90220 Oulu, Finland; 2 Research Unit of Health Sciences and Technology, Department of Radiology, Oulu University Hospital, Oulu University, 90220 Oulu, Finland; 3 Research Service Unit, Research Unit of Clinical Medicine, Oulu University Hospital, 90220 Oulu, Finland; 4 Department of Pediatric Surgery and Pediatric Urology, University Children’s Hospital of Tübingen, 72076 Tübingen, Germany

**Keywords:** distal forearm fractures, pediatric, non-operative treatment, radiographic follow-up

## Abstract

Distal forearm fractures are common in children and are usually treated nonoperatively. No consensus has been reached on how to perform clinical and radiographic follow-up of these fractures. Our aim was to study whether radiographic and clinical follow-up is justified. We included 100 consecutive patients with non-operatively treated distal forearm fractures who were treated at Oulu University Hospital in 2010–2011. The natural history of the fractures during the nonoperative treatment was analyzed by measuring the potential worsening of the alignment during the follow-up period. The limits of acceptable fracture position were set according to the current literature using “strict” or “wide” criteria for alignment. We determined the rate of worsening fracture position (i.e., patients who reached the threshold of unacceptable alignment). In relation to splinting, we evaluated how many patients benefited from clinical follow-up. Most of the fractures (98%) preserved acceptable alignment during the entire follow-up period when wide criteria were used. The application of stricter criteria for alignment in radiographs showed loss of reduction in 19% of the fractures. Worsening of the alignment was recognized at a mean of 13 days (range 5–29) after the injury. One in three (32%) patients needed some intervention due to splint loosening or failure. Radiographic follow-up of nonoperatively treated distal forearm fractures remains questionable. Instead, clinical follow-up is important, as 32% of patients needed their splints fixed.

## 1. Introduction

Distal radius fractures are the most common fractures in children, accounting for 20–30% of all pediatric fractures [1,2,3,4,5,6]. The highest incidence rates are observed in older school-age boys (10–16 years) [3,4,7,8] due to a pubertal growth spurt, when the skeleton is weaker, and increased physical activity [7]. Fractures of the distal radial physis and metaphysis show a great capacity for spontaneous correction of injury-related deformity [9,10,11,12]; however, the remodeling capacity depends on the location of the fracture and the child’s age. Fractures near the physis in young children have the greatest potential to remodel. Most pediatric distal forearm fractures can be treated nonoperatively [13] by casting or splinting [14] with or without a closed reduction. Loss of alignment of distal radius fractures after nonoperative treatment is common, with a rate reported between 7% and 91% [13,15,16,17,18]. Fracture-related and treatment-related risk factors for secondary displacement are presented in Table 1.

Much controversy exists regarding the acceptable degree of angular deformity [29,30]. Up to 30–35° of angular deformity in the sagittal plane and 10° in the coronal plane are suggested for adequate remodeling if the patient has at least 5 years of growth left [11,31].

Conventional radiographs of children’s nonoperatively treated forearm fractures are usually taken 1 and 2 weeks after the injury and again at the time of completion of immobilization (4–5 weeks) [17,32] to evaluate the alignment. During this follow-up, the clinician also assesses the condition of the splint.

The aim of the present study was to determine the clinical importance of radiographic follow-up of pediatric distal forearm fractures after splinting. We hypothesized that less than 20% of children with a distal forearm fracture treated with non-operative means would show worsening of the alignment or increasing displacement that would meet the wide reduction criteria. Another aim was to determine the importance of clinical follow-up in detecting the need for splint repairing. We hypothesized that more than 50% of the patients would require splint correction or renewal.

## 2. Materials and Methods

This study included 100 children less than 16 years old who were consecutively treated for distal forearm fracture in the pediatric orthopedic outpatient clinic of Oulu University Hospital, Finland from January 2010 to December 2011. Acute distal radius fractures, with or without distal ulna fractures, were included if they were considered suitable for nonoperative treatment. Only fractures with the potential for displacement were accepted. For the coherence of the study patients, we excluded a few patients who had circumferential casts and included only patients treated with a splint. Stable buckle fractures and patients who were referred for operative treatment were excluded. The fracture types were classified according to the AO pediatric comprehensive classification of long bone fractures [33]. All clinical and radiological data concerning the injuries in question were retrieved from hospital medical records.

Most children with acute fractures, including forearm fractures, receive primary treatment in the emergency room at Oulu University Hospital. The treating physician can be a family doctor or a surgeon. The conservative treatment protocol includes the application of a splint with or without manipulation. The reduction, if needed, is performed under local or general anesthesia. We mainly use a custom-made below-elbow padded dorsal or volar splint of plaster of Paris, which is curved to the other side of the forearm to give three-point support. The splint reaches distally to the metacarpophalangeal joint. Post-reduction radiographs are taken before discharge. The first follow-up visit is scheduled 7–10 days later and includes a radiograph and evaluation by a pediatric orthopedic surgeon, a pediatric surgeon familiarized with childhood trauma or a resident in these specialties. Subsequent visits with radiographs usually occur at 2 weeks and 4 weeks following the injury. Undisplaced or minimally displaced fractures may have only one follow-up visit with radiographs at 1–2 weeks after the injury without further actions. Follow-up is decided on an individual basis for every patient, and no obligatory follow-up schema is applied for these patients.

In accordance with the study protocol, the patients’ radiographic and clinical follow-up findings were studied at every follow-up visit. The radiographs included anteroposterior and lateral projections taken via a digital radiograph system. The angulation degree was defined as the angle between the central longitudinal axis of the proximal and distal fragments of the distal radius. The anterior–posterior and lateral displacement was expressed in millimeters. One researcher (MP), with 20 years of experience in pediatric radiology and childhood trauma imaging, performed all radiographic analyses.

Two criteria, “strict” and “wide” (Table 2), were defined according to the current literature [11,12,13,19,29,34,35] prior to the study to evaluate the course of the nonoperatively treated distal forearm fractures and their alignment. The 2 criteria included the upper limits for the angulation in the anterior–posterior and coronal directions and for translational displacement in any direction, expressed in millimeters. We adjusted for the age and gender of the patients regarding these criteria (Table 2). Institutional approval for the study was obtained prior to its initiation (21 October 2019, 146/2009).

The main outcome of the study was a worsening fracture alignment beyond the accepted limits (Table 2) during the follow-up period. In total, 2 separate analyses were performed to analyze the worsening alignment in relation to the 2 limits of acceptance (wide and strict). As a secondary outcome, the importance of clinical follow-up was determined based on the frequency of splint repair or new casting at every follow-up visit.

### Statistical Analysis

Based on prior prospective studies of Asadollahi et al. and Alemdaroğlu et al. [18,36], we hypothesized that 20% of the fractures would lose their alignment during follow-up. An odds ratio of 10 for the loss of alignment of children’s distal radial fractures was considered clinically significant when using strict criteria. This was reasonable given that the occurrence of re-displacement in this self-controlled case study was 2% among non-operative children. With a statistical power of 80% and a two-sided α error of 0.05, we calculated that the required sample size was 87 children. To overcome the potential missing data of the individual consecutive cases in this research, we conducted a sample size analysis prior to the study initiation, and we opted for the study population size of 100 to exceed the minimum sample size. The quantitative data are described as the mean and range for categorical variables and as frequencies and proportions with 95% confidence intervals constructed using the exact Clopper–Pearson method. Basic statistics were calculated using IBM SPSS Statistics for Windows, Version 27 (Armonk, NY, USA, IBM Corp.) statistical software. Confidence intervals of the proportions were calculated using StatsDirect Statistical Software, version 3.3.5 (Wirral, England, StatsDirect Ltd. 2008).

## 3. Results

### 3.1. Study Sample and Patients’ Characteristics

Altogether, 100 patients were included in the study. Of these, one patient received their primary treatment at another hospital, but their follow-up visits were carried out at the study institution. Of the original one hundred thirteen patients, thirteen patients were excluded from the analysis. Overall, five of these thirteen patients did not have post-reduction radiographs for follow-up, one patient had a primary fracture that was missed, and one patient with a Salter–Harris type 1 fracture was excluded because the fracture was one of a kind. The other six patients were excluded because they were treated with a circumferential cast.

The mean age of the patients was 11.7 years (range 4–16 years). Overall, 70% of the patients were aged 11–16 years, and 60% of the patients were male. The most common injury cause (73%) was a fall from a standing height. Snowboarding, downhill skiing, scootering, and skateboarding were the most common recreational activities related to distal forearm fractures in this population. The radius fractures comprised 18% complete metaphyseal fractures, 32% incomplete metaphyseal fractures, and 50% physeal Salter–Harris type 2 fractures. The fracture types, according to the AO pediatric comprehensive classification of long bone fractures, were 23r-E/2, 23r-M/2, and 23r-M/3. The baseline characteristics of the patients, their injury types, and their treatments are presented in Table 3.

Altogether, 226 follow-up visits with radiographs took place. Follow-up visit one occurred 2–10 days after the injury in 95 patients. The second follow-up visits took place between 11 and 20 days (41 patients) and the third between 21 and 41 days (83 patients) after injury. Only seven patients had a later follow-up visit, occurring between 42 and 212 days after the injury.

In terms of reduction, 55% of the fractures were reduced on admission and 44% were immobilized without reduction. The fracture alignment after the primary treatment is presented in Table 4.

### 3.2. Occurrence of Loss of Alignment

A total of 84 (84%) of the fractures showed acceptable alignment after primary treatment according to the strict criteria, and 99 (99%) fractures were acceptably aligned according to the wide criteria. The only case exceeding the wide criterion of coronal angulation after primary treatment was an 11-year-old girl, who had a complete metaphyseal fracture. Most (97/99) of the fractures (98%, 95% confidence interval [CI] 93–100%) showed acceptable alignment during the entire follow-up period according to the wide criteria (Table 5).

**Figure 1:**
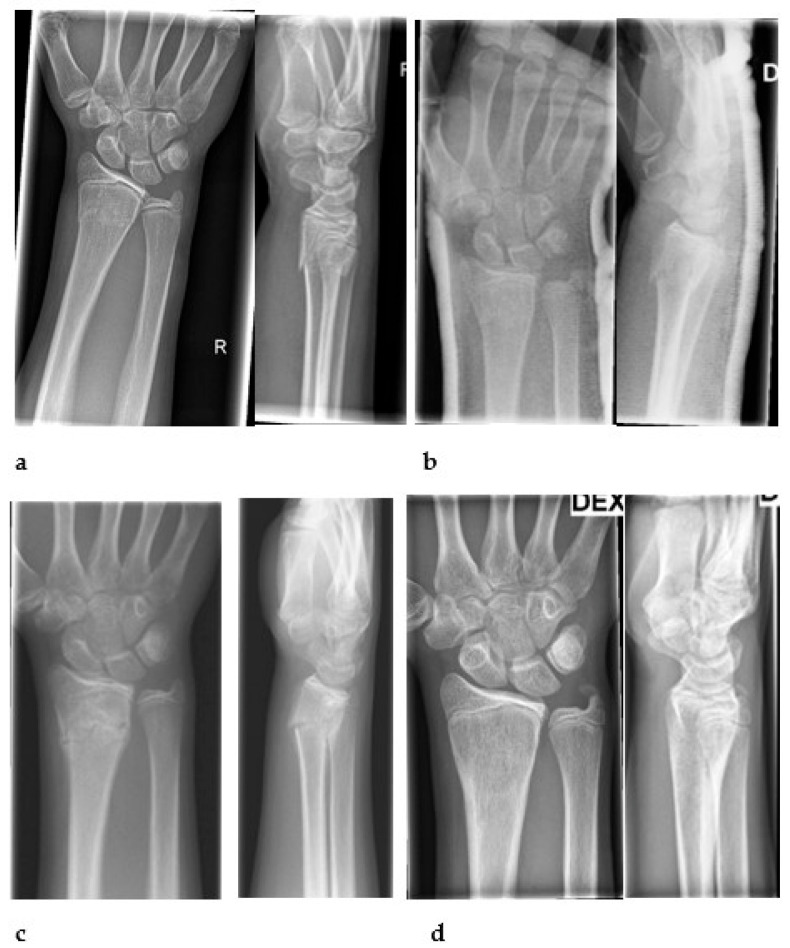
A 14-year-old girl injured her right wrist in downhill skiing. A complete metaphyseal fracture was detected and treated by immobilization without manipulation (**a**). In a follow-up visit at one-week mark, the alignment was good (**b**), but four weeks later a worsening alignment in radius was revealed with 24° dorsal angulation (**c**). A six-month follow-up visit was arranged showing good fracture position (**d**).

In total, two patients dropped out during the follow-up period; these were two girls aged 12 and 14 years with complete metaphyseal fractures. Deterioration of the sagittal alignment in the radial fractures was detected 4 weeks after the injury (Figure 1).

Altogether 16/84 (19%, 95% CI 11–29%) of the patients lost acceptable alignment during the follow-up when the strict criteria were used. According to the different fracture types, loss of alignment occurred in 18% (9/50) of the SH 2 fractures, 13% (4/32) of the incomplete metaphyseal fractures, and 17% (3/18) of the complete metaphyseal fractures. Worsening of the alignment was recognized at a mean of 13 days (range 5–29).

### 3.3. Rate of Reduction during Follow-Up

In total, one patient of the 100 (1%, 95% CI 0.03–5%) was re-treated during the follow-up period. She was a 12-year-old girl who had a complete metaphyseal fracture with dorsal angulation (17°), and she was primarily treated with immobilization without manipulation. At 12 days after the injury, the angulation was similar, but the first reduction was determined. However, the fracture malalignment of 17° was within the limit of acceptable alignment, and no intervention was needed according to the wide criteria.

### 3.4. Need for Splint Repair

The clinical evaluation of the patients resulted in repairs of splints in 32 (32%, 95% CI 23–42%) of the cases. Most of the cases (24, 24%) required repairs within 10 days after injury, whereas seven casts were fixed at the second follow-up visit. One patient received a removable splint 3 weeks after the trauma. The intervention with the splint was a consequence of loosening or failure of the splint, irritative reactions, or other inconvenience caused by the splint.

## 4. Discussion

Distal radius fractures are the most common pediatric fractures, but due to their good remodeling capacity, they can mostly be treated nonoperatively [13]. No consensus has yet been reached regarding the limits of acceptable angular and translational displacement for decisions on whether to reduce the fracture or to leave correction of the position to remodeling during growth. Therefore, in this study, we retrospectively used two different criteria, “strict” and “wide”, to evaluate the course of 100 consecutive pediatric nonoperatively treated distal forearm fractures and their alignments. Instead, we decided not to use the (re-)reduction as the primary variable of treatment failure because it depends on the particular decision of each particular treating physician rather than on the evidence-based guidelines for this injury. We found that 16/84 (19%) of the fractures showed secondary displacement beyond the acceptable alignment when the strict criteria were used. When the wide criteria were applied, only two (2%) of ninety-nine fractures benefited from radiographic follow-up. Maccagnano et al. have evaluated accordingly predetermined radiographic parameters for failure predictors of conservative treatment in pediatric forearm shaft fractures in their study [37].

Consideration of the benefit of radiographic follow-up in accordance with the patient’s age depends on how much remodeling potential is available should a displacement occur [29,38]. Van der Sluijs found that a malunion of 15° had a mean remodeling time of 12 months, and this increased to 36 months for a malunion of 30° [39]. In metaphyseal fractures, a mean remodeling rate of 2.5° per month and up to 7.6° of correction per month has been reported in children [40]. A positive correlation between the remodeling speed and malunion angulation and a negative correlation between remodeling speed and remodeling time have been found [40,41]. The coronal remodeling potential in distal radius fractures of skeletally immature patients is remarkable. A mean coronal angulation remodeling rate of 2.3° per month was detected by Lynch et al. in 2020 among 36 pediatric patients aged from 4 to 12 years [35]. In Australia, Roth et al. studied 66 children suffering from nonoperatively treated distal forearm fractures that had failed and undergone re-displacement: 24 were re-manipulated, and 42 were left to heal in the position of the angular deformity. Re-manipulation of the distal forearm fractures in the children < 12 years did not improve their outcomes [34]. Therefore, Roth et al. suggest the following acceptance criteria when re-angulation occurs: up to 30° of angulation in children < 9 years, 25° of angulation in children aged 9–12 years, and 20° of angulation in children ≥ 12 years. They also encourage clinicians to be more reluctant to perform re-reductions. A Norwegian prospective study reported that 12 patients out of 88 showed more than 15 degrees of malangulation after nonoperative treatment. Of these twelve, seven participated for 7 years of follow-up visits and demonstrated complete remodeling [42]. Similar long-term outcomes have been found among children under 10 years of age independent of residual fracture deformity during fracture healing [12,20,23,35].

In addition to the displacement criteria and the patient’s maturity, the age and location of the fracture, any clinical deformity, and the clinicians’ judgement can influence the treatment options [18]. Complete fracture displacement [13,18], translation of more than half the bone diameter, volar angulation, and a simultaneous ulnar fracture at the same level as the radius fracture have been identified as predictors of instability [36]. In their study, Asadollahi et al. reported a secondary displacement rate of 28.8%, but only 7.4% of the patients needed a second procedure [18]. Their criteria for displacement were even tighter than the strict criteria used in the present study. Notably, the use of strict radiographic displacement criteria alone may increase the number of unnecessary interventions and follow-up visits for fractures with a high potential to remodel. An internet-based survey conducted by Bernthal et al., (2015) on the management of pediatric distal radius fractures revealed that, in addition to the fracture displacement and the patient’s age, the surgeon’s subspecialty and the practice environment affected the treatment recommendations. Pediatric orthopedic surgeons favored the most conservative management [30].

In the present study, 32 (32%) of the patients needed clinical interventions during follow-up, namely fixing or renewing the splint. In most of these cases (24/32; 75%) the need arose in the first 10 days after the injury. The need to fix a splint can be the result of the splint loosening when post-traumatic edema in soft tissues decreases. Some of the required clinical interventions may have been due to the quality of the splint and the experience of the personnel in the emergency department, which is not dedicated to treating children’s fractures. This finding supports the idea of routine clinical follow-up of distal forearm fracture patients during the early follow-up period. An option for later clinical assessment could be to interview the patient and the parent(s) by phone and, if necessary, to the arrange an outpatient clinic visit. Routine clinical follow-up could be performed by trained orthopedic nurses without intervention by a surgeon.

Because of the high frequency of pediatric distal radius fractures, the expenses related to the treatment of these fractures are not trivial. Godfrey et al. [43] retrospectively investigated the treatment-related costs of closed distal radius fractures in 5640 children treated at a large academic children’s hospital in the USA. The median costs of closed reductions without manipulation, closed reductions in the emergency department or radiology procedure suite, and percutaneous pinning in the operating room were USD 1390, USD 4263, and USD 9389, respectively. This included only medical services. The fracture management approach and use of the operating room had the greatest influence on the treatment costs. They concluded that choosing not to perform a closed reduction in patients whose fractures will adequately remodel during their growth could reduce treatment costs. Considering the primary treatment of distal radial metaphyseal fractures, Orland et al. [44] estimated that in children younger than 10 years of age 27% of closed reduction procedures were unnecessary. The costs of closed reduction and manipulation using procedural sedation in the emergency department were estimated to be eight times higher than the costs of cast immobilization in an outpatient clinic. Cost saving of up to 12% of the total cost of nonoperatively treated pediatric forearm fractures can be achieved by eliminating the radiographic follow-up visit at 4 weeks after the injury, according to Luther et al. [32].

Health care resources could be optimized by employing wide criteria for acceptable alignment in the primary treatment of children’s distal forearm fractures, favoring splinting/casting in an outpatient clinic without manipulation, and conducting rigorous casting. As most activity concerning loose, unfit, or broken splints appeared in the one follow-up visit at 1 week after the injury, clinical inspection at the one-week mark seems justifiable. Informing the patient and the parents about the good natural course of fracture healing, even with moderate displacement, and encouraging them to contact the treating unit with any concerns about splint impairment will increase compliance.

### Strengths and Limitations

The retrospective nature of this study did not produce systematic long-term outcomes. The number and timing of follow-up visits were also inconsistent in the study population. We do not have available information for all risk factors for the displacement of distal forearm fractures. Because no widely accepted guidelines are available concerning acceptable alignment of children’s distal forearm fractures, the effect of the treating physician’s preferences may have influenced the treatment choices. Although we included 100 fractures, 70% of the patients were aged 11–16 years and the number of patients in the younger age groups was quite small. However, the risk for loss of reduction is higher in children ≥ 11 years old. Accordingly, when the fractures were classified by fracture type, only 18 patients had fractures with the greatest potential for dislocation (i.e., complete metaphyseal fractures). A strength of this study is its design with an objective measurable outcome, namely the malalignment beyond a predetermined threshold, despite the dependence of the rate of (re-)reduction on the decision by the treating surgeon for every single patient.

## 5. Conclusions

We evaluated 100 consecutive nonoperatively treated distal forearm fractures with the potential to displace. One in five of the fractures (16/84, 19%) that were acceptable after primary treatment were found to lose reduction when strict criteria for reduction were applied. Worsening alignment was found in two patients (2%) with complete metaphyseal fractures when the wide criteria were applied. However, only one of the one hundred fractures underwent a reduction during follow-up in real life. The use of strict criteria for acceptable alignment would have increased the number of fracture reductions and follow-up visits with radiographs. Nevertheless, this study does not resolve whether these potential (re-)reductions would have been important in achieving any long-term benefits regarding the clinical outcomes of the patients. Another important finding was that one in three of the patients needed their splint repaired, and this mostly occurred 1 week after the injury.

## Figures and Tables

**Table 1:** Risk factors for secondary displacement of nonoperatively treated distal forearm fractures in children.

Fracture-related	Complete/high-grade initial displacement ^1^
	Both-bone fracture ^2^
	Obliquity of the fracture line ^3^
	Initial angulation and shortening ^4^
Treatment-related	Quality of reduction ^5^
	Three-Point Index ^6^
	Cast Index ^7^
	Padding Index ^7^
	Canterbury Index ^7^
Patient-related	Age ≥ 11 years ^8^
	Obesity ^9^

References: ^1^ [13,18,19,20,21]; ^2^ [19]; ^3^ [20]; ^4^ [21]; ^5^ [18,21,22,23,24,25]; ^6^ [20]; ^7^ [26]; ^8^ [27]; ^9^ [28].

**Table 2:** Limits of acceptable alignment according to the “strict” and “wide” criteria.

		Sagittal Plane		Coronal Plane	Displacement
Treatment Protocol	Age	Boys	Girls		
“Strict”					
	0–5	>25°	>25°	>10°	>10 mm
	6–10	>20°	>20°	>10°	>10 mm
	≥11	>15°	>10°	>5°	>5 mm
“Wide”					
	0–5	>35°	>35°	>20°	>10 mm
	6–10	>30°	>30°	>15°	>10 mm
	≥11	>25°	>20°	>15°	>10 mm

**Table 3:** Baseline characteristics of the patients, their injury types, and treatments.

		*n* = 100
Age, years		11.7 ± 2.8
Age distribution		
	0–5 years	3
	6–10 years	27
	11–16 years	70
Gender		
	males	60
	females	40
Fracture side		
	left	70
	right	30
Type of injury		
	Fall	73
	Fall > 1 m	12
	Traffic incident	3
	other	12
Fracture type		
	Salter–Harris II	50
	Incomplete metaphyseal	32
	Complete metaphyseal	18
Treatment *		
	Immobilization in situ	44
	Reduction with LA **	43
	Reduction with GA ***	12
Treating physician on admission *		
	Family doctor	57
	Junior Surgeon	39
	Senior Surgeon	3
Time of treatment		
	Day, 6 a.m.–9 p.m.	72
	Night 9 p.m.–6 a.m.	28

* *n* = 99, ** Local anesthesia (LA), *** General anesthesia (GA).

**Table 4:** Fracture position during primary care.

Treatment on Admission	Immobilization In Situ (*n* = 44)	Reduction (*n* = 55)	Post Reduction (*n* = 55)
Angulation on sagittal plane *	8.6	22.9	5.4
range	0–17	5–57	0–18
Angulation on coronal plane *	0.5	5.2	0.7
range	0–10	0–26	0–16
Displacement **	0.5	5.1	1.4
range	0–5	0–24	0–7

* Mean, degrees; ** mean, millimeters.

**Table 5:** Fracture alignment after primary care and during follow-up visits No 1–4.

Protocol	Alignment	Primary Care	Visit 1	Visit 2	Visit 3	Visit 4
Strict	Accepted	84	88	36	79	7
	Not accepted	16	7	5	4	0
Wide	Accepted	99	95	41	81	7
	Not accepted	1	0	0	2	0
Current practice *		100	95	41	83	7

* True number of patients at every visit.

## Data Availability

Research data available on request from the authors.
